# UNDERSTANDING TRANSPORT NEEDS OF OLDER AUSTRALIAN DRIVERS: SUSTAINABLE AND SMART INITIATIVES FOR SAFE MOBILITY

**DOI:** 10.1093/geroni/igae098.1577

**Published:** 2024-12-31

**Authors:** Jennie Oxley, Judith Charlton, Sjaan Koppel, David Logan, Selby Coxon

**Affiliations:** Monash University, Melbourne, Victoria, Australia; Monash University, Melbourne, Victoria, Australia; Monash University, Melbourne, Victoria, Australia; Monash University, Melbourne, Victoria, Australia; Monash University, Melbourne, Victoria, Australia

## Abstract

Access to adequate and appropriate transport options enables older people to continue as thriving community participants, to reach services and to maintain social connections. While transport needs are diverse, and tend to change over time, there is little information on current and future transport patterns, and the awareness, acceptance and adoption of new technologies. A national online survey was administered to current drivers in Australia. A sample of 705 drivers provided information on available travel modes and use of these modes, awareness of in-vehicle technologies and future use of vehicle technologies. The findings revealed high use of private vehicles, walking and taxis but little use of other travel modes (bicycles, motorcycles, rideshare, community services and public transport). Age, gender and residential location influenced the availability and use/potential use of some transport options. Overall awareness of in-vehicle technologies was generally low and particularly so amongst older and female participants. There was some appetite to use emerging technologies in the future. The findings inform the development of effective strategies and initiatives aligned with healthy ageing and wellbeing targets, increased sustainability, resilience and connectedness, creation of healthier travel choices and healthier environments to promote acceptance and use of a range of transport options and uptake of safer vehicles equipped with in-vehicle technologies to ultimately enhance safe and sustainable mobility of older road users. Such strategies may be particularly useful to support extended safe driving amongst older adults with chronic medical conditions, such as driver monitoring/alert systems.

